# Supplementary material for: Comparative Study of Gut Microbiota in Wild and Captive Giant Pandas (Ailuropoda melanoleuca)
Source: Genes (Basel). 2019 Oct 20;10(10):827. doi: 10.3390/genes10100827 (PMC6826394; doi:10.3390/genes10100827)
Supplement: Supplementary file 1 [file genes-10-00827-s001.zip › supplementary materials/Table S3.doc]

**Table S3. Summary of whole-genome shotgun (WGS) sequencing.**

| **Sample ID** | **Sequencing platform** | **Raw reads** | **Host and bamboo removed reads** | **Dropping the single reads** | **Final retained paired-end clean reads** |
| --- | --- | --- | --- | --- | --- |
| WP1 | Illumina Hiseq 2500 | 10,073,426 × 2 | 15,740 × 2 | 820,393 × 2 | 9,237,293 × 2 |
| WP3 | Illumina Hiseq 2500 | 16,048,282 × 2 | 15,158 × 2 | 4,293,699 × 2 | 11,739,425 × 2 |
| WP4 | Illumina Hiseq 2500 | 12,399,937 × 2 | 7,165 × 2 | 466,132 × 2 | 11,926,640 × 2 |
| WP5 | Illumina Hiseq 2500 | 17,111,625 × 2 | 46,191 × 2 | 4,768,487 × 2 | 12,296,947 × 2 |
| WP10 | Illumina Hiseq 2500 | 11,696,066 × 2 | 18,056 × 2 | 295,165 × 2 | 11,382,845 × 2 |
| WP12 | Illumina Hiseq 2500 | 12,196,026 × 2 | 25,832 × 2 | 445,122 × 2 | 11,725,072 × 2 |
| WP14 | Illumina Hiseq 2500 | 12,204,632 × 2 | 8,745 × 2 | 411,679 × 2 | 11,784,208 × 2 |
| JX | Illumina Hiseq 2500 | 11,733,103 × 2 | 83,407 × 2 | 2,314,564 × 2 | 9,335,132 × 2 |
| HH | Illumina Hiseq 2500 | 11,477,287 × 2 | 506,186 × 2 | 447,562 × 2 | 10,523,539 × 2 |
| DL1 | Illumina Hiseq 2500 | 11,245,869 × 2 | 308,986 × 2 | 438,119 × 2 | 10,498,764 × 2 |
| FY1.27 | Illumina Hiseq 2500 | 11,245,869 × 2 | 677,037 × 2 | 493,722 × 2 | 11,233,949 × 2 |
| ZM2.1.27 | Illumina Hiseq 2500 | 12,611,226 × 2 | 91,620 × 2 | 476,480 × 2 | 12,043,126 × 2 |
| WG | Illumina Hiseq 2500 | 12,489,755 × 2 | 257,460 × 2 | 2,011,437 × 2 | 10,220,858 × 2 |
| GZ1.9.2 | Illumina Hiseq 2500 | 11,944,071 × 2 | 208,976 × 2 | 412,976 × 2 | 11,322,119 × 2 |
