# Supplementary figures and images for: Comparative Study of Gut Microbiota in Wild and Captive Giant Pandas (Ailuropoda melanoleuca)
Source: Genes (Basel). 2019 Oct 20;10(10):827. doi: 10.3390/genes10100827 (PMC6826394; doi:10.3390/genes10100827)

Figure S1

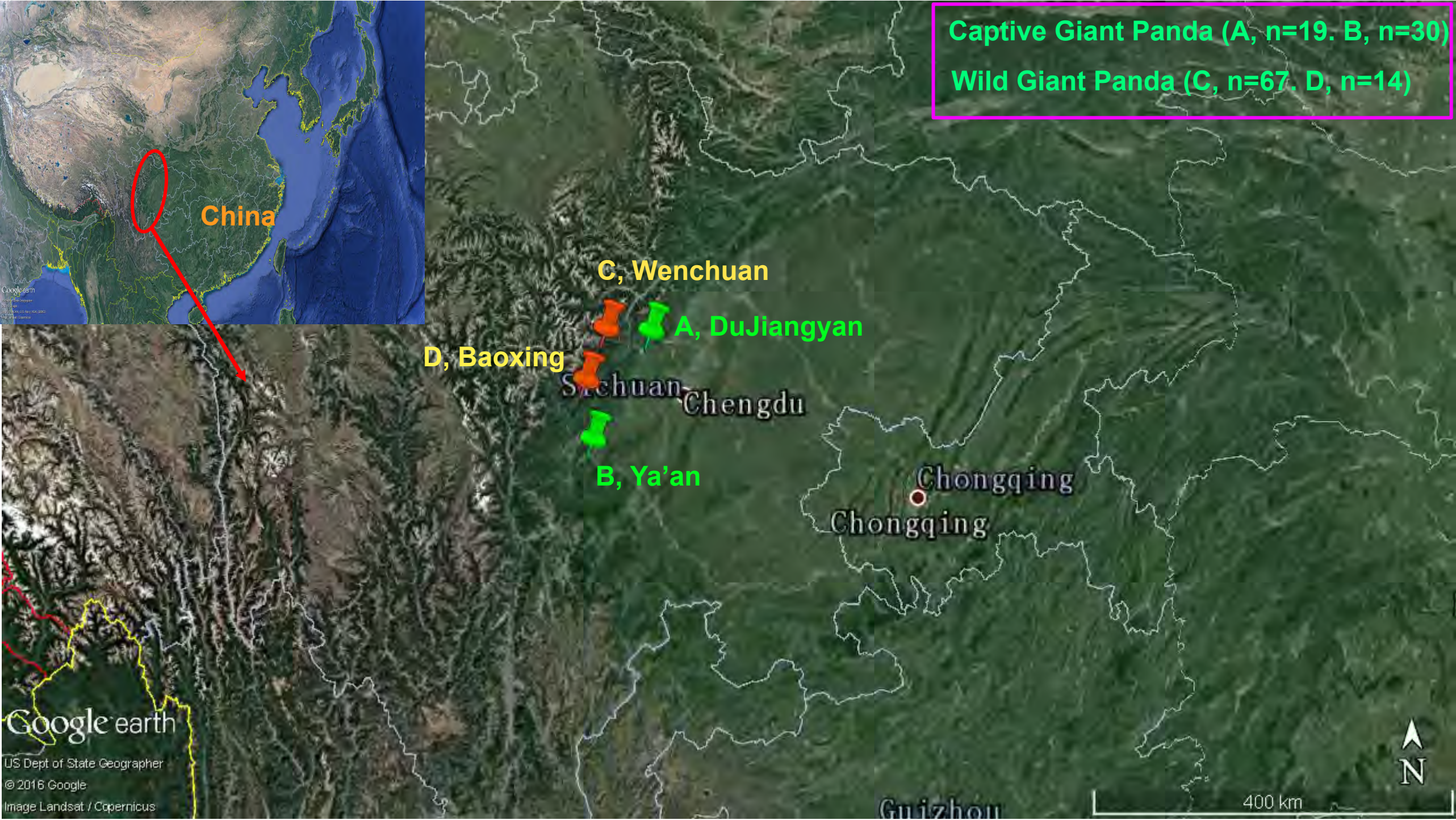

Supplement: Supplementary file 1 [file genes-10-00827-s001.zip › supplementary materials/Figure.S1.pdf]

Figure.S2

**A**

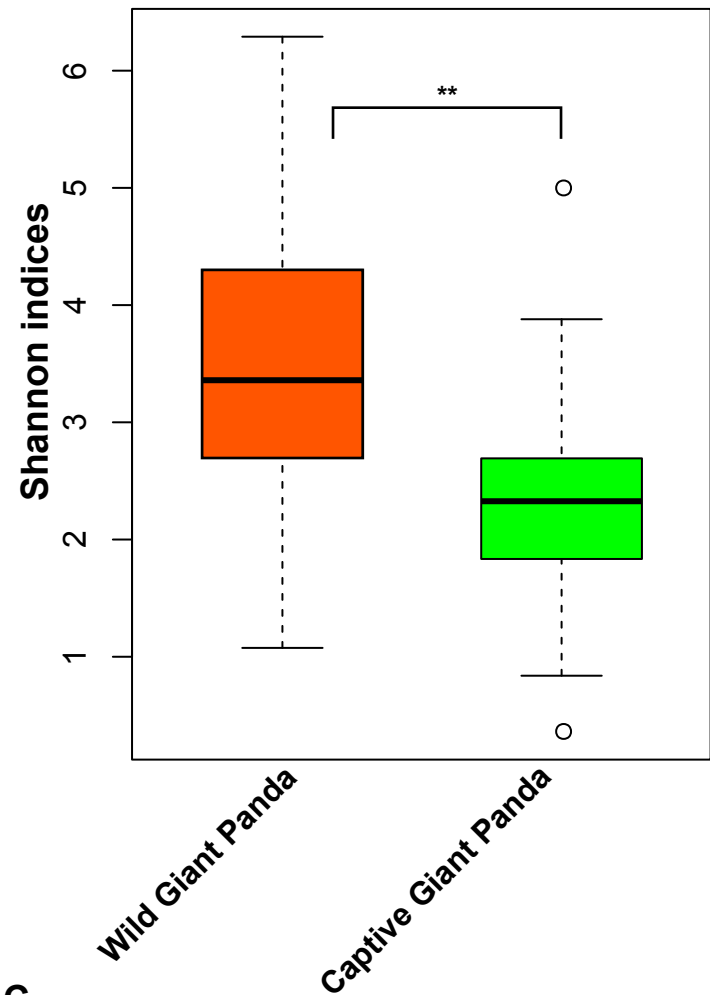

**B**

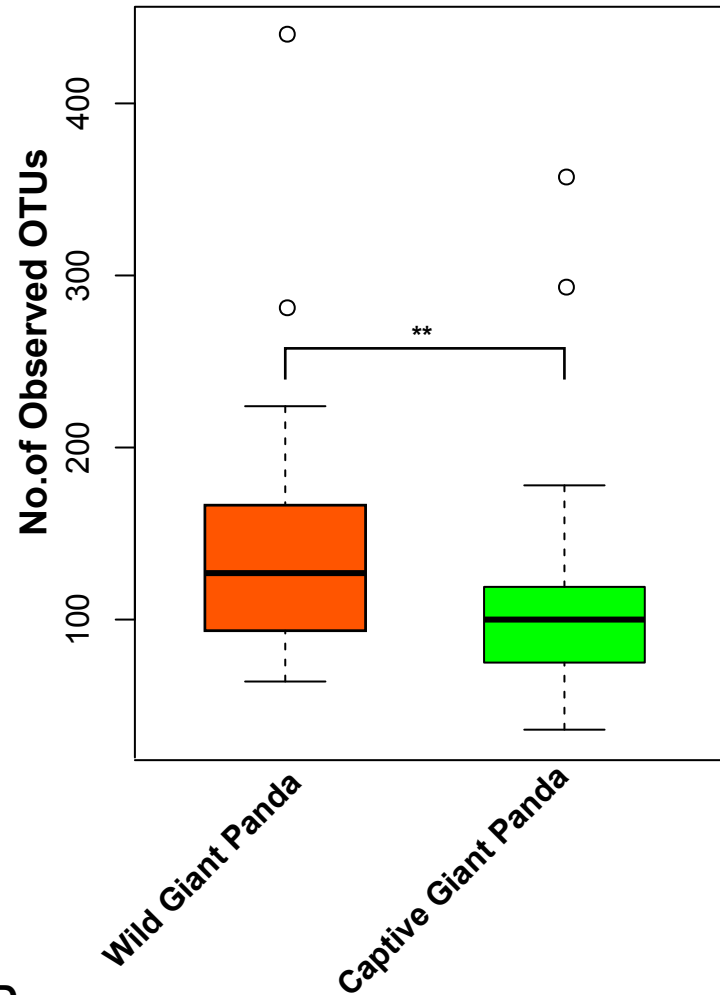

**C**

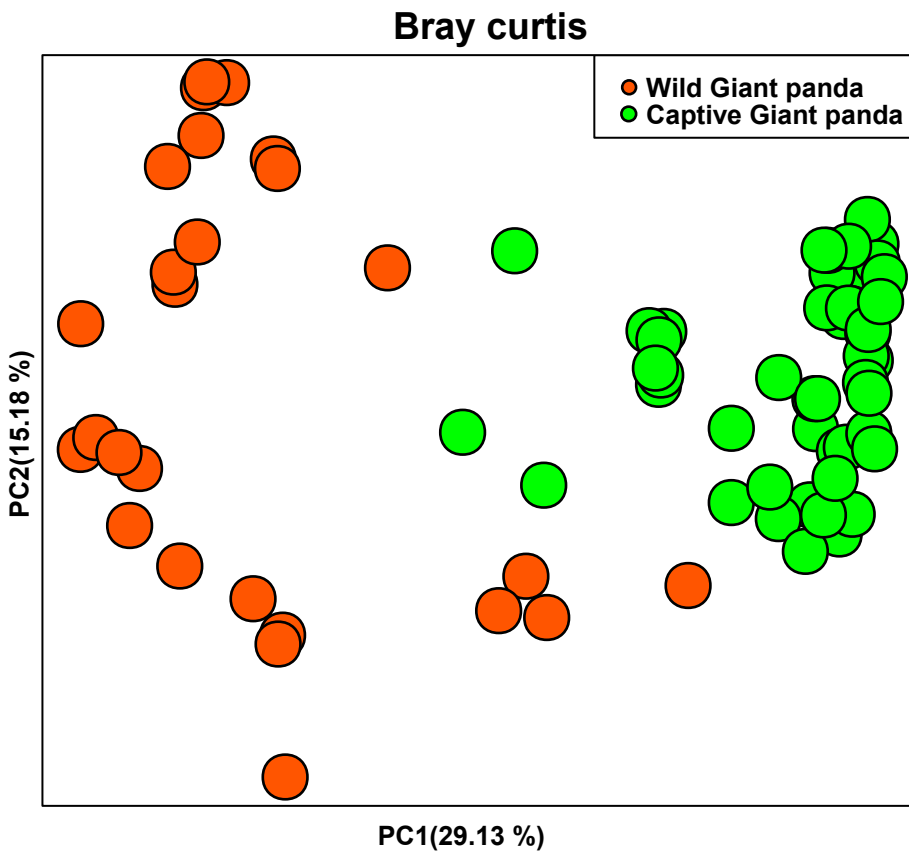

**D**

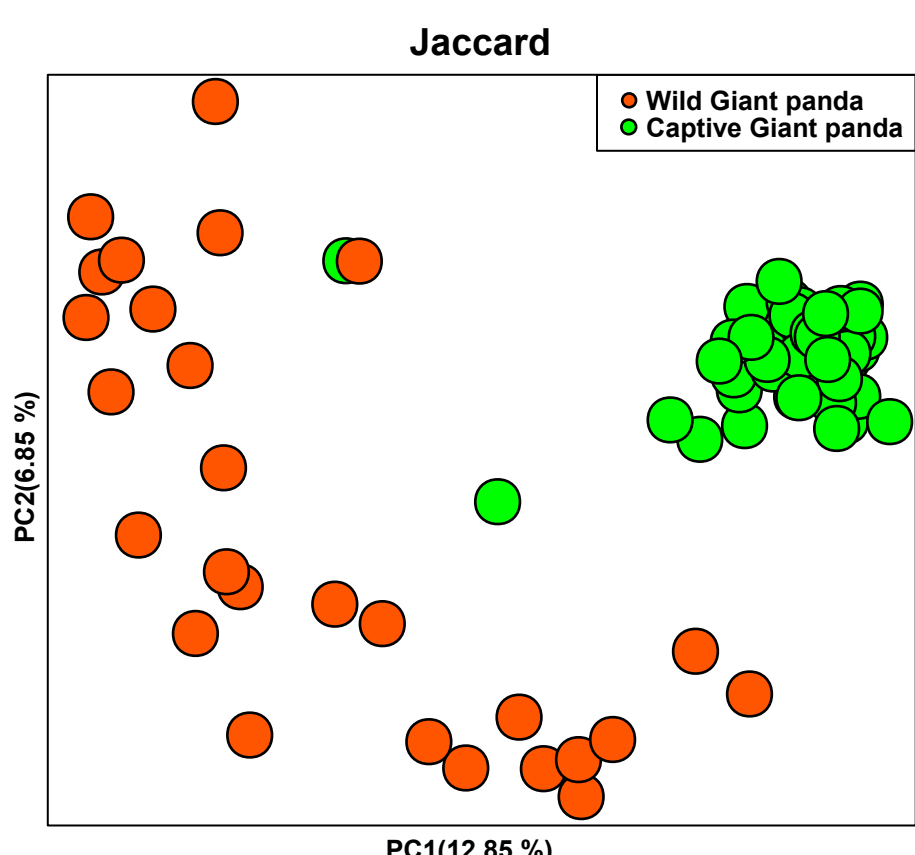

Supplement: Supplementary file 1 [file genes-10-00827-s001.zip › supplementary materials/Figure.S2.pdf]

Figure S3

A

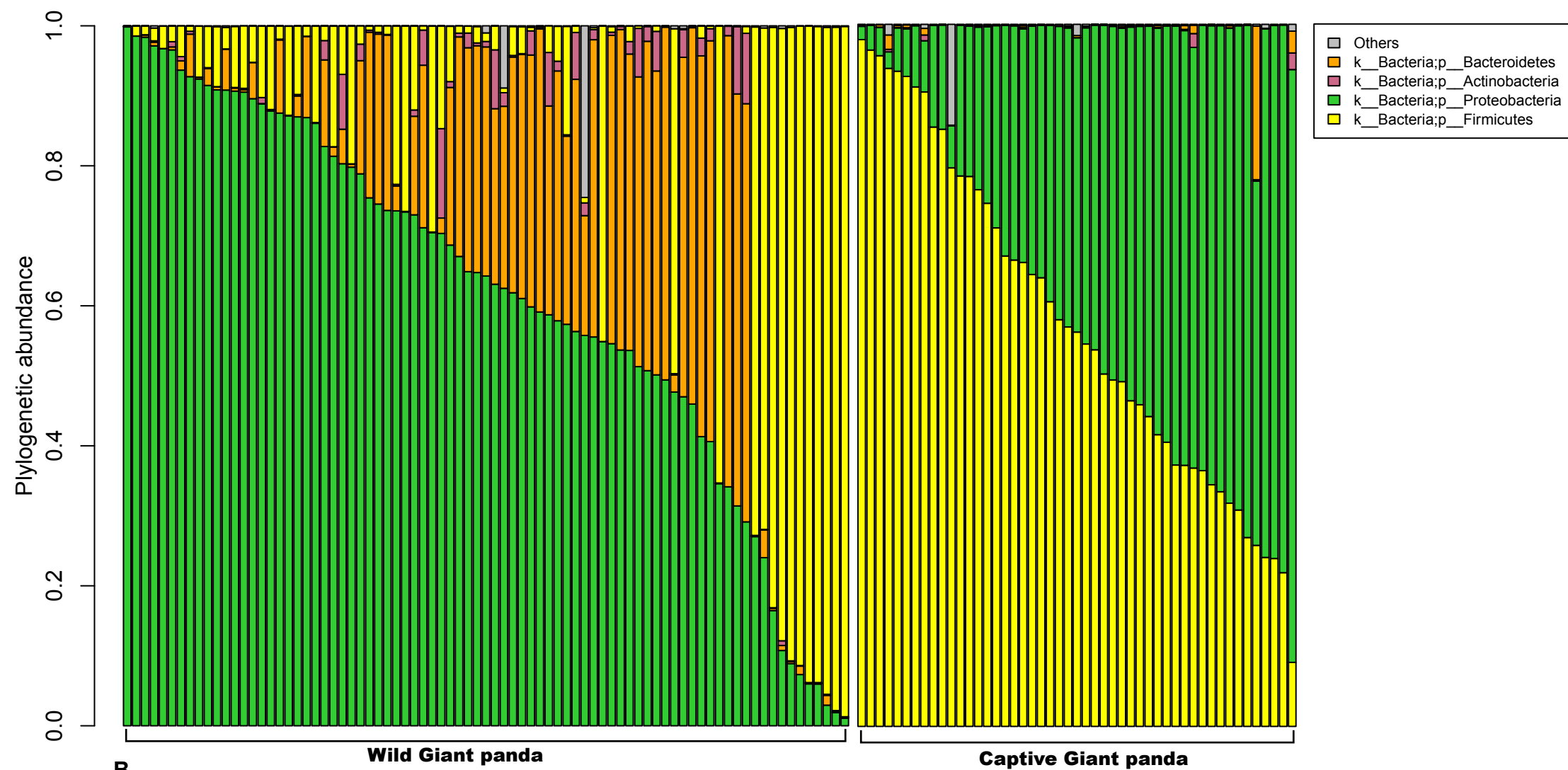

B

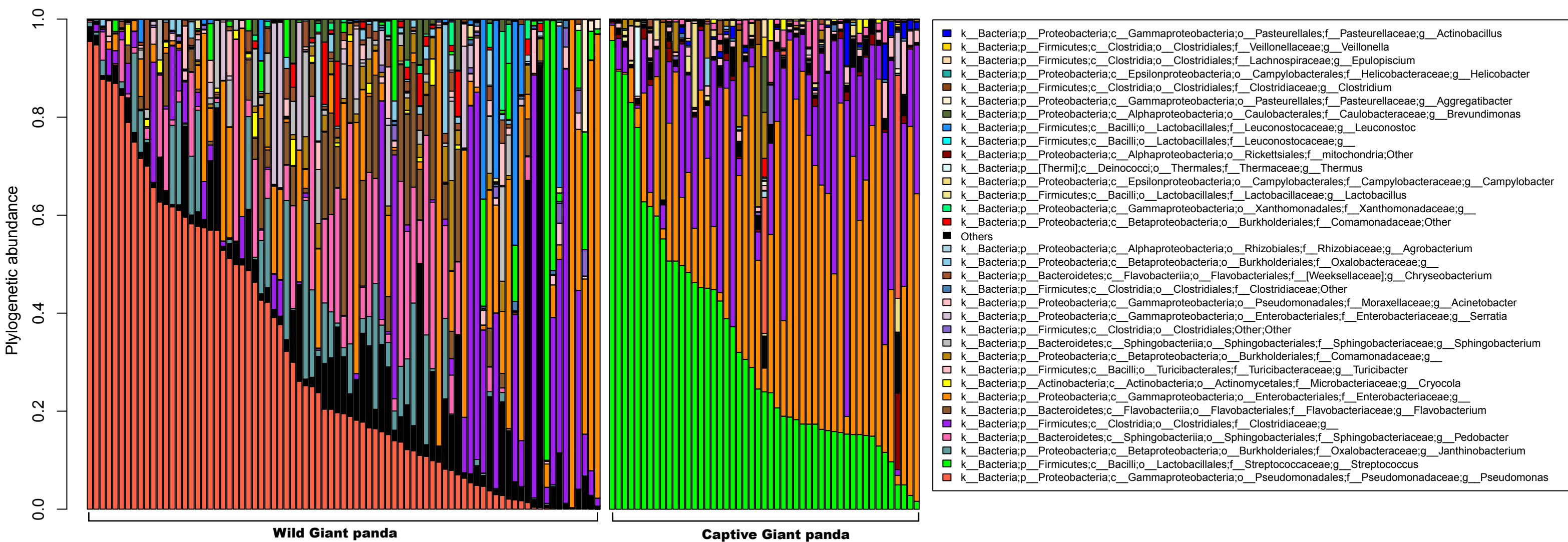

Supplement: Supplementary file 1 [file genes-10-00827-s001.zip › supplementary materials/Figure.S3.pdf]

Figure S4

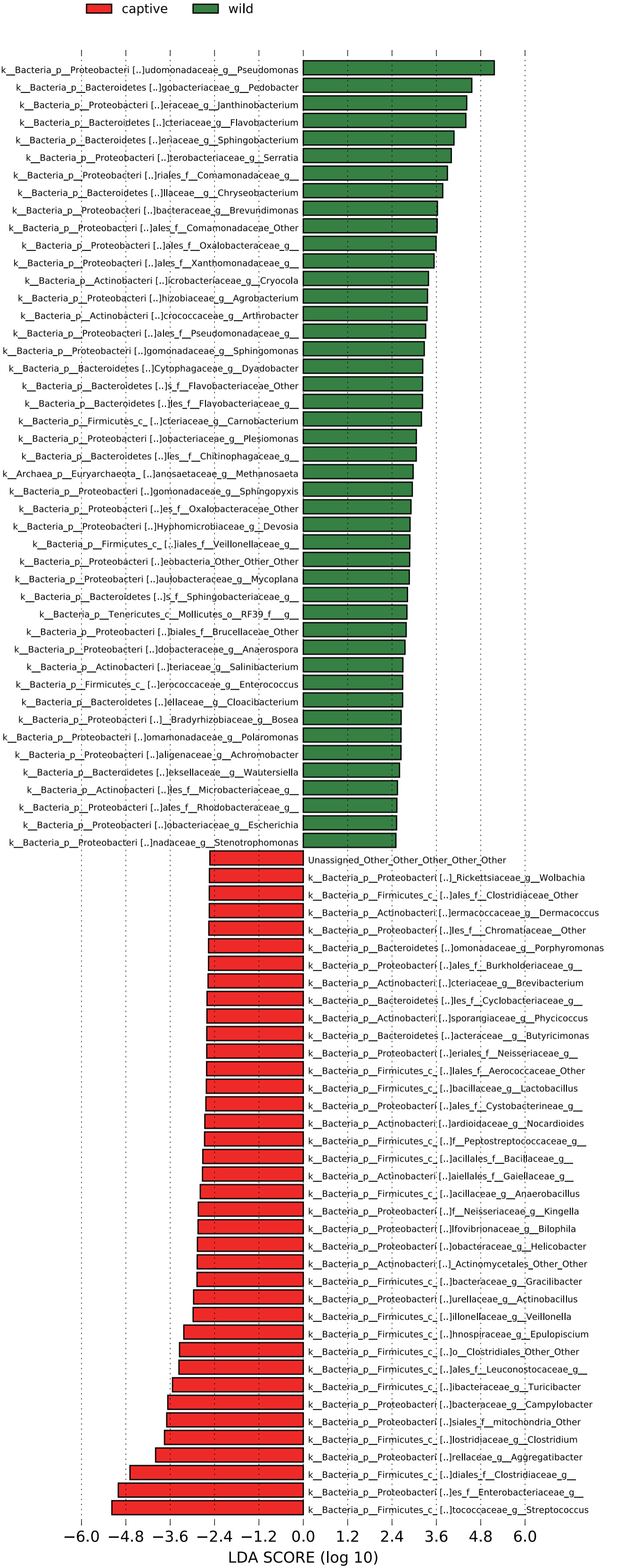

Supplement: Supplementary file 1 [file genes-10-00827-s001.zip › supplementary materials/Figure.S4.pdf]

Figure S5

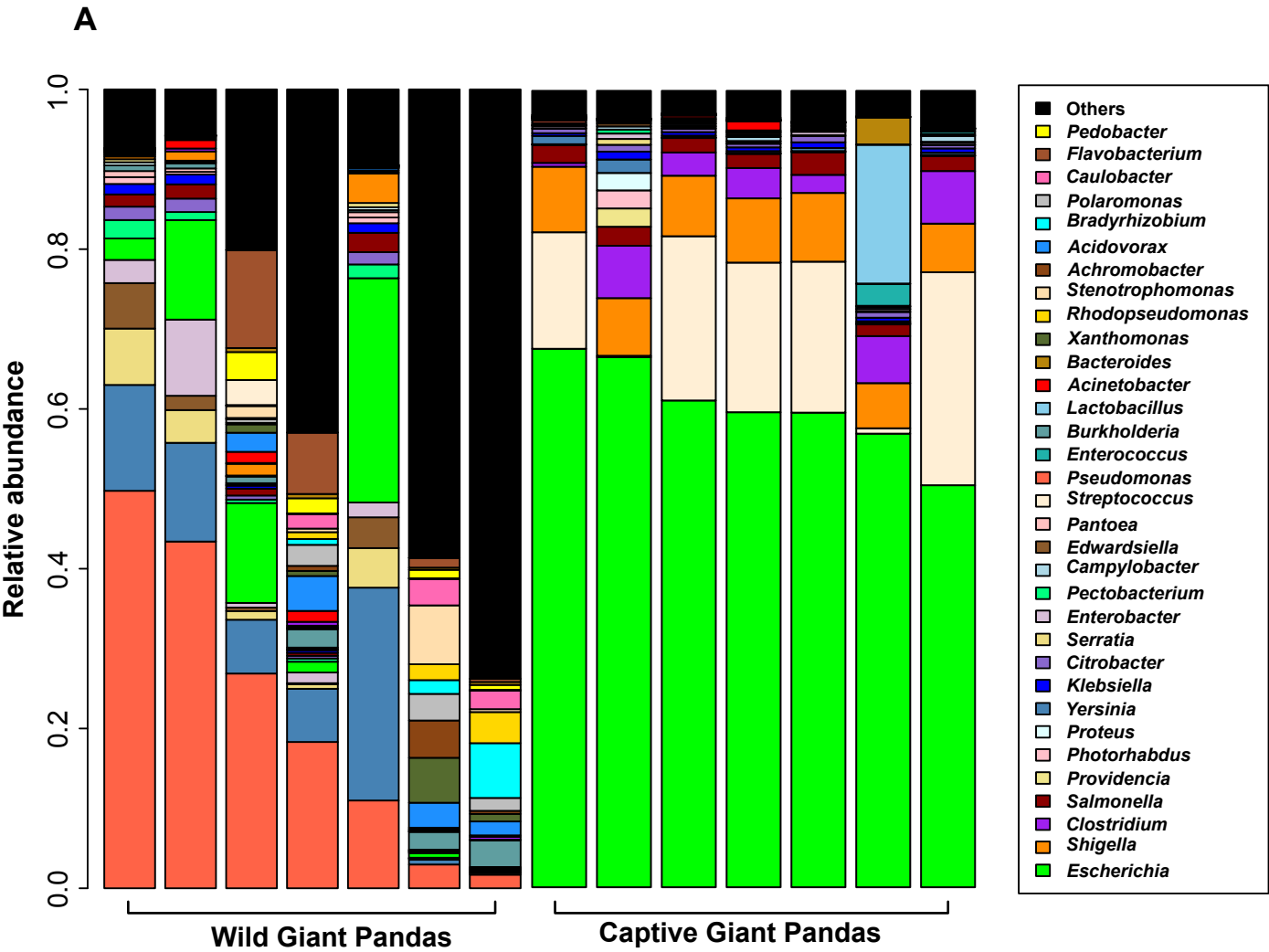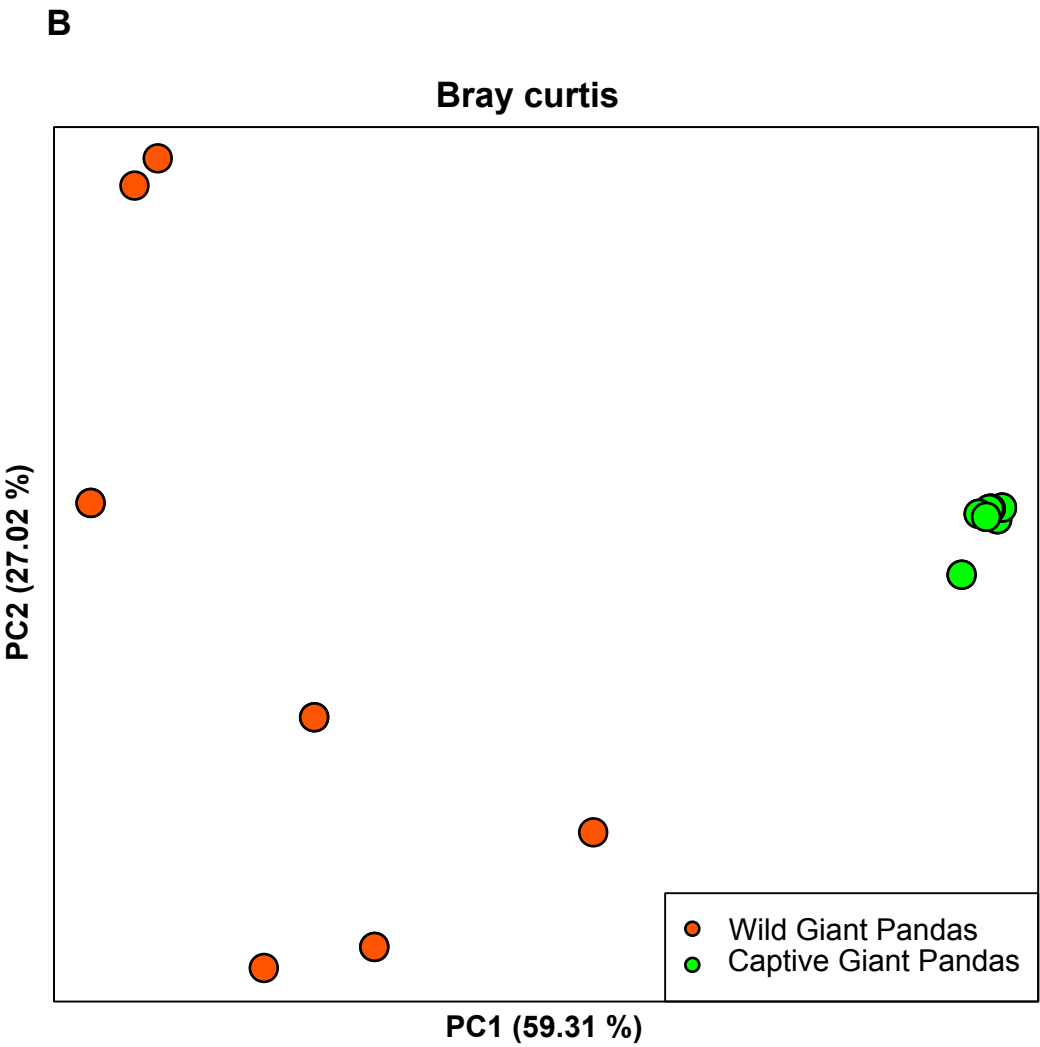

Supplement: Supplementary file 1 [file genes-10-00827-s001.zip › supplementary materials/Figure.S5.pdf]

Figure S6

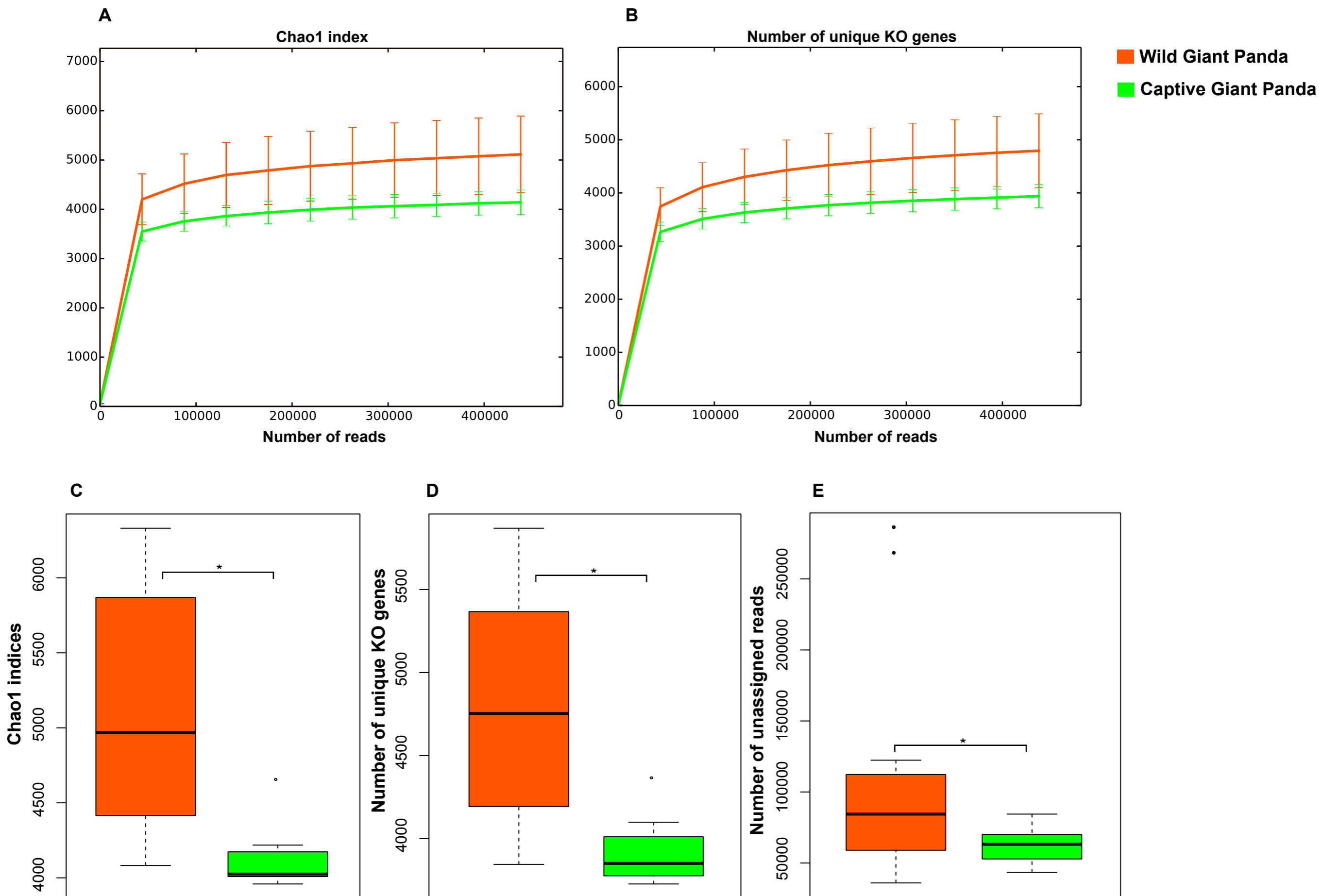

Supplement: Supplementary file 1 [file genes-10-00827-s001.zip › supplementary materials/Figure.S6.pdf]

A

Jaccard

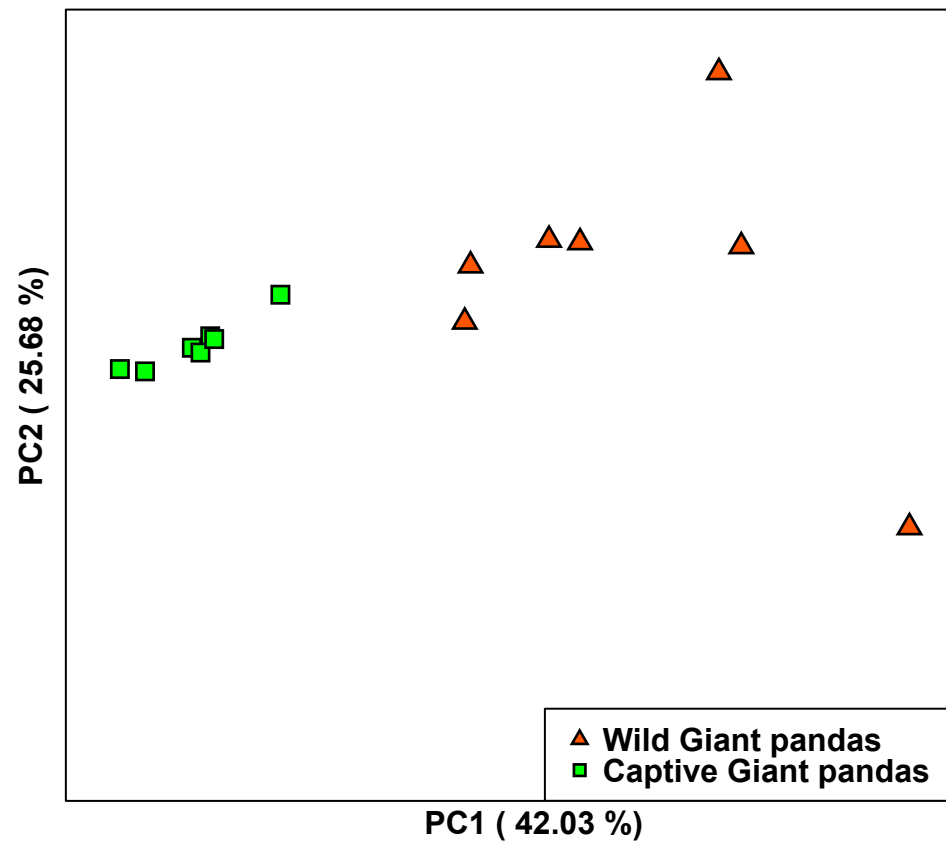

B

Bray-Curtis

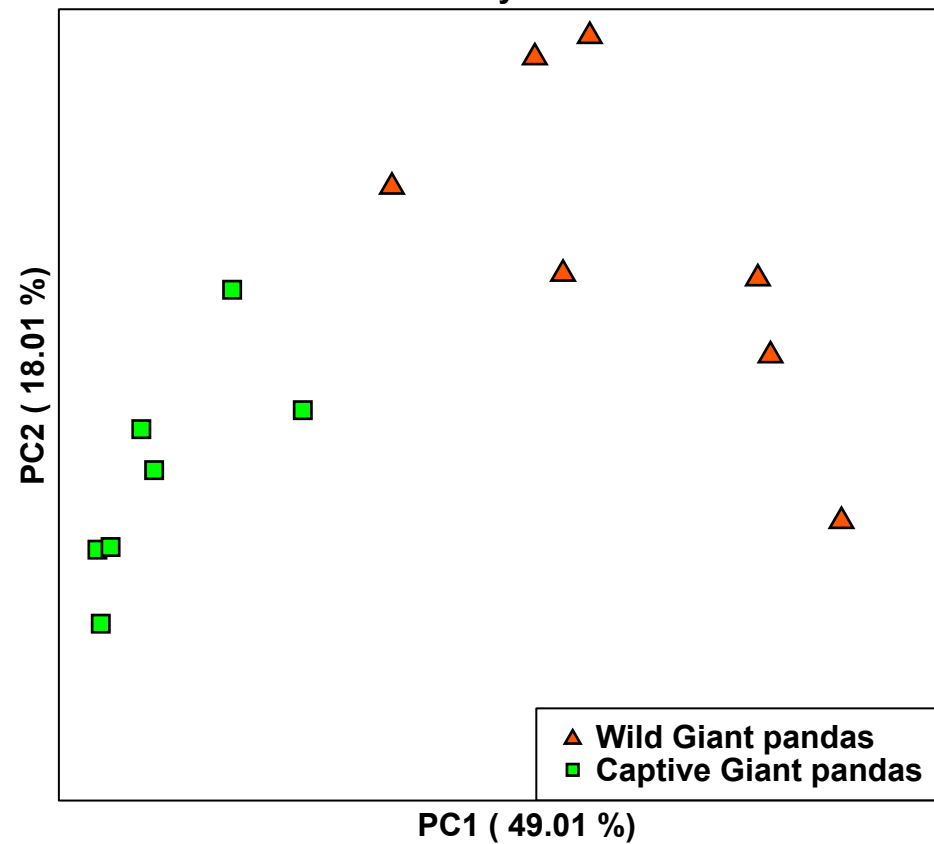

C

Endoglucanase (EC:3.2.1.4)

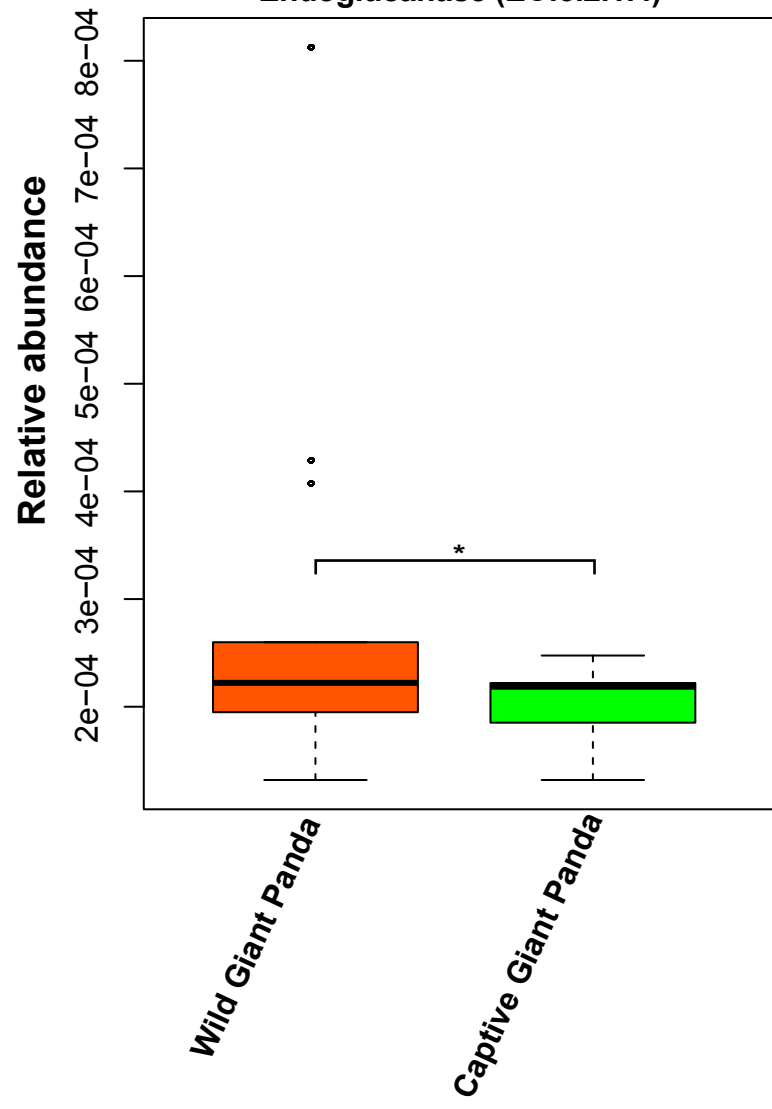

D

Alpha amylase (EC:3.2.1.1)

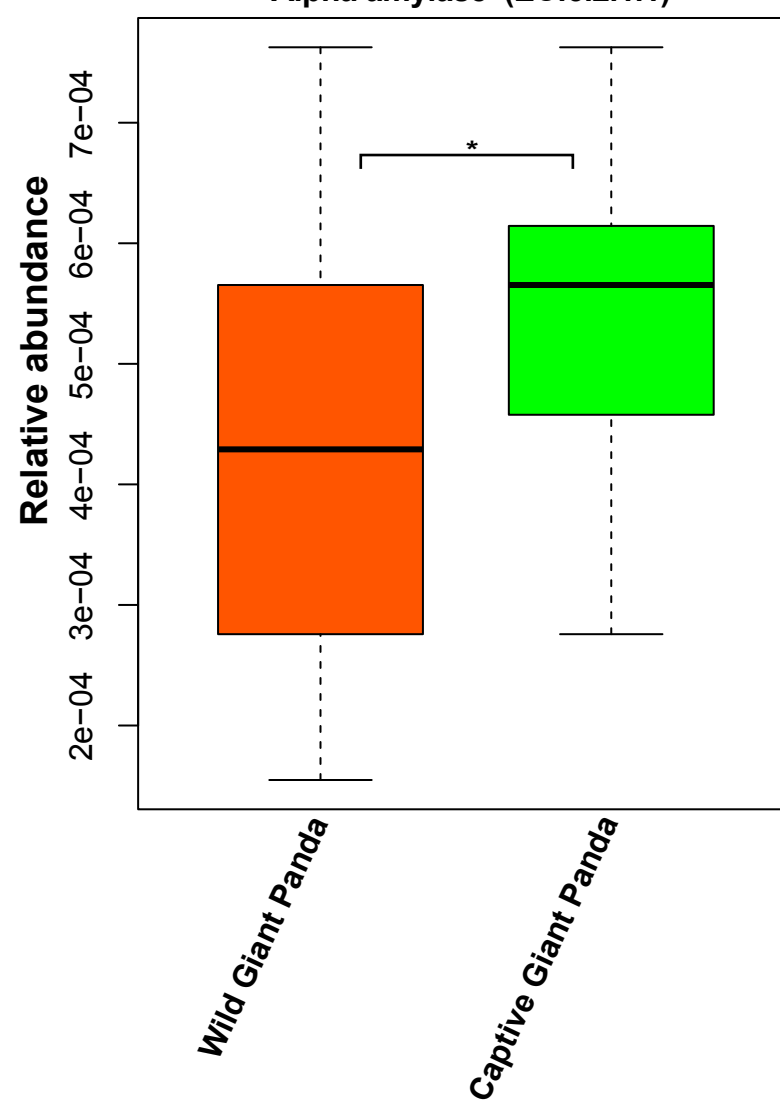

Supplement: Supplementary file 1 [file genes-10-00827-s001.zip › supplementary materials/Figure.S7.pdf]

Figure S8

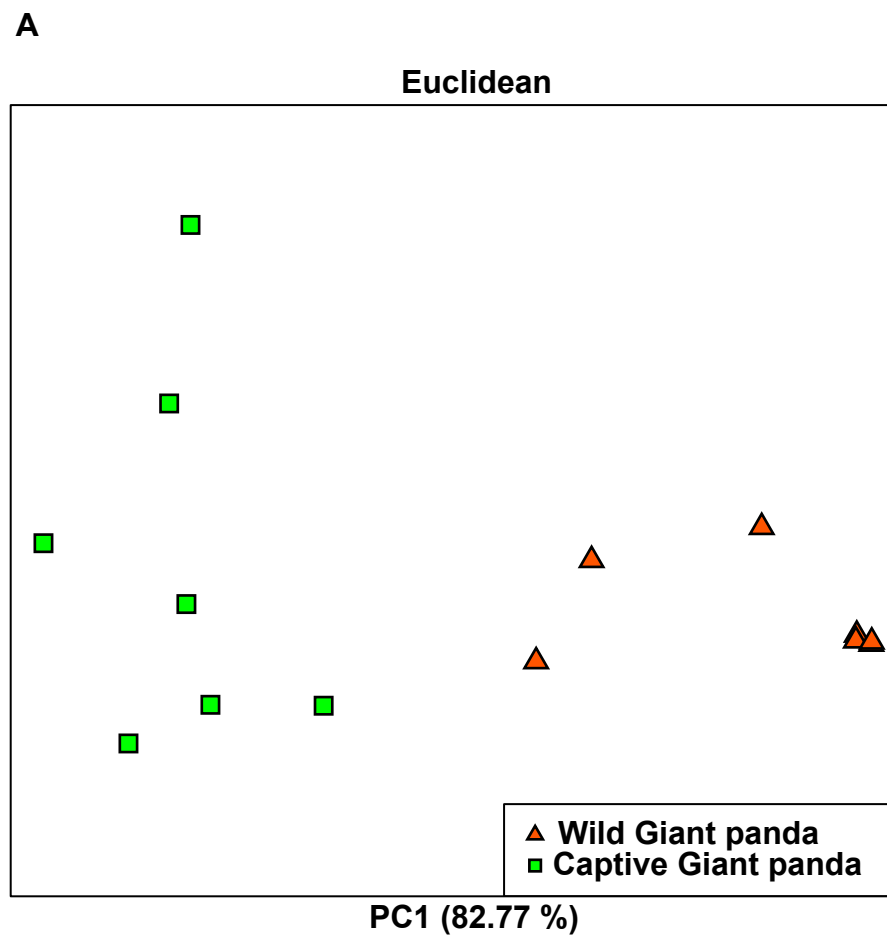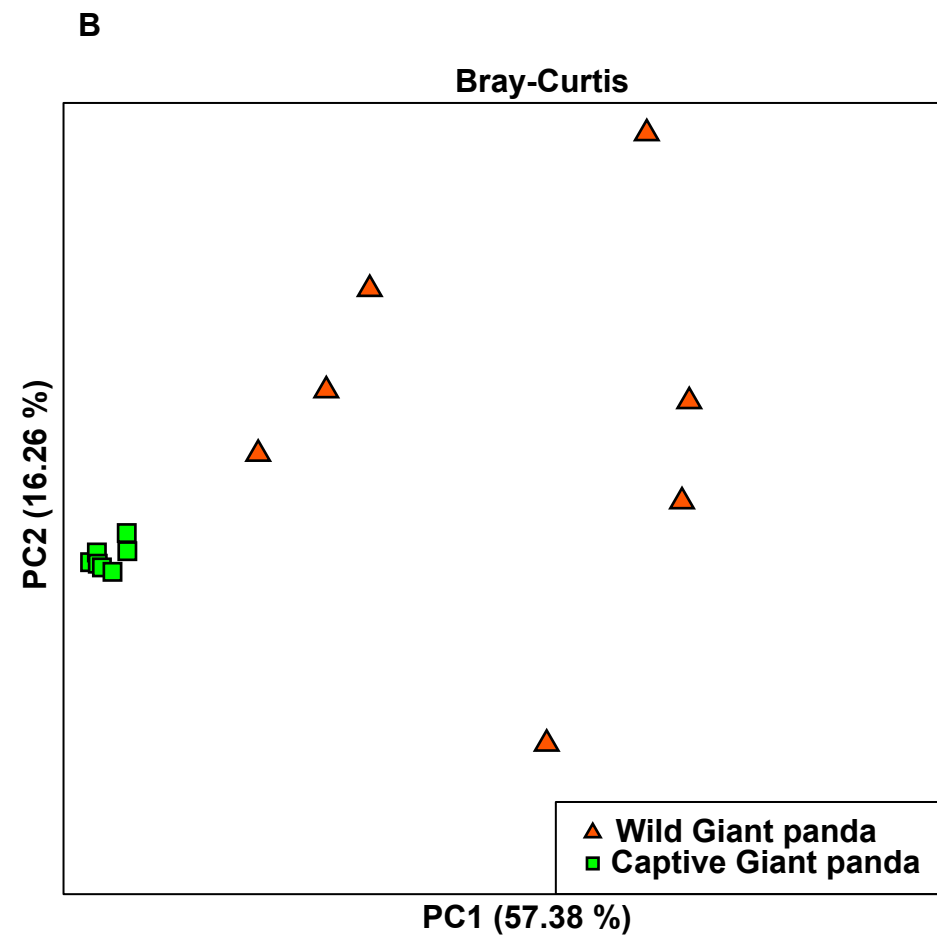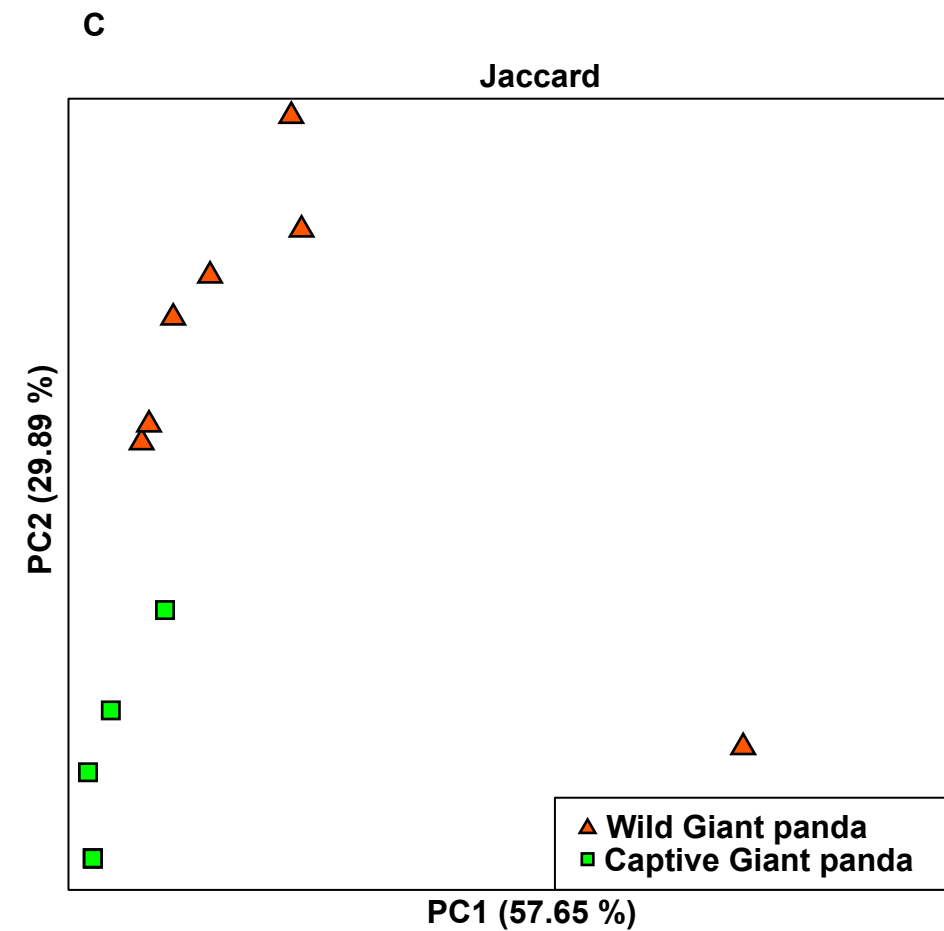

Supplement: Supplementary file 1 [file genes-10-00827-s001.zip › supplementary materials/Figure.S8.pdf]
