# Supplementary material for: Comparative Study of Gut Microbiota in Wild and Captive Giant Pandas (Ailuropoda melanoleuca)
Source: Genes (Basel). 2019 Oct 20;10(10):827. doi: 10.3390/genes10100827 (PMC6826394; doi:10.3390/genes10100827)
Supplement: Supplementary file 1 [file genes-10-00827-s001.zip › supplementary materials/Supplemental file legends.doc]

**Additional files**

**Figure S1.** The geographical distribution of panda groups sampled in this study.

**Figure S2.** Comparison of alpha and beta diversity of the gut microbiome of wild (Randomly selecting based on the DNA analysis. The Shannon diversity index (A) and number of observed OTUs (B); Principal Coordinates Analysis plots of Bray-Curtis (C) and Jaccard (D) distances.

**Figure S3.** Stacked bar plots illustrate the mean relative abundance of OTUs at the phylum (A) and genus (B, top 20 OTUs) level in the fecal microbiomes of wild and captive giant pandas.

**Figure S4.** Bacterial taxa (based on genus level) significantly differentiated among wild and captive giant pandas by LEfSe. (P-value cut-off of 0.01 and a minimum effect size of 2.5).

**Figure S5.** Comparison of composition and beta diversity between wild and captive giant pandas based on metagenome datas. (A) The relative abundance of OTUs at genus level; (B) Principal Coordinates Analysis plots of Bray-Curtis distance.

**Figure S6.** Functional diversity of gut metagenomes in wild and captive giant pandas. Rarefaction curves illustrating (A) the Chao1 index and (B) the number of unique KO genes. Box and whisker plots illustrating (C) the Chao1 index, (D) the number of unique KO genes, and (E) the number of unassigned reads. *p < 0.05 (Mann-Whitney U)

**Figure S7.** Functional differences in the fecal microbiomes of wild and captive giant pandas. (A) Jaccard and (B) Bray-Curtis distance matrices were computed on KEGG gene counts and visualized in PCoA plots. (C) The relative abundance of the endoglucanase gene in wild and captive pandas. (D) the relative abundance of the alpha amylase gene in wild and captive pandas. *p < 0.05 (Mann-Whitney-U).

**Figure S8.** Antibiotic Resistance Profiles of wild and captive pandas. Distinct profiles were assessed by computing (A) Euclidean, (B) Bray-Curtis, and (C) Jaccard distances and visualized in PCoA plots.

**Table S1.** Detailed information of sampling and sequencing of 16S rDNA gene in this study.

**Table S2.** The representative sample for wild giant pandas based on the DNA analysis..

**Table S3.** Summary of whole-genome shotgun (WGS) sequencing.

**Table S4.** PERMANOVA pseudo-F and p-values associated with specific factor in the wild giant pandas.

**Table S5** The medical history of captive giant pandas which were selected for metagenome sequencing.
