# Supplementary material for: Comparative Study of Gut Microbiota in Wild and Captive Giant Pandas (Ailuropoda melanoleuca)
Source: Genes (Basel). 2019 Oct 20;10(10):827. doi: 10.3390/genes10100827 (PMC6826394; doi:10.3390/genes10100827)
Supplement: Supplementary file 1 [file genes-10-00827-s001.zip › supplementary materials/Table S2.doc]

**Table S2. The representative sample for wild giant pandas based on the DNA analysis.**

| **ID Number** | **Sample 1** | **Sample 2** | **Sample 3** | **Sample 4** | **Randomly selecting the representative sample** |
| --- | --- | --- | --- | --- | --- |
| ID 1 | wild3 | wild8 | wild17 | wild72 | wild17 |
| ID 2 | Wild4 |  |  |  | wild4 |
| ID 3 | wild7 |  |  |  | wild7 |
| ID 4 | wild9 | wild30 | wild40 | wild66 | wild66 |
| ID 5 | wild10 |  |  |  | wild10 |
| ID 6 | wild13 | wild33 | wild74 |  | wild33 |
| ID 7 | wild14 | wild18 | wild25 |  | wild25 |
| ID 8 | wild16 | wild38 | wild67 | wild75 | wild75 |
| ID 9 | wild19 |  |  |  | wild19 |
| ID 10 | wild24 | wild34 |  |  | wild24 |
| ID 11 | wild26 | wild27 |  |  | wild27 |
| ID 12 | wild36 | wild71 |  |  | wild71 |
| ID 13 | wild39 | wild45 | wild69 |  | wild39 |
| ID 14 | wild41 |  |  |  | wild41 |
| ID 15 | wild42 |  |  |  | wild42 |
| ID 16 | wild46 |  |  |  | wild46 |
| ID 17 | wild47 |  |  |  | wild47 |
| ID 18 | wild51 |  |  |  | wild51 |
| ID 19 | wild52 |  |  |  | wild52 |
| ID 20 | wild56 |  |  |  | wild56 |
| ID 21 | wild59 |  |  |  | wild59 |
| ID 22 | wild63 |  |  |  | wild63 |
| ID 23 | wild64 |  |  |  | wild64 |
| ID 24 | wild65 |  |  |  | wild65 |
| ID 25 | wild68 |  |  |  | wild68 |
| ID 26 | wild15 |  |  |  | wild15 |
| ID 27 | wild76 |  |  |  | wild76 |

ID
