# Supplementary material for: Comparative Study of Gut Microbiota in Wild and Captive Giant Pandas (Ailuropoda melanoleuca)
Source: Genes (Basel). 2019 Oct 20;10(10):827. doi: 10.3390/genes10100827 (PMC6826394; doi:10.3390/genes10100827)
Supplement: Supplementary file 1 [file genes-10-00827-s001.zip › supplementary materials/Table S4.doc]

**Table S4. PERMANOVA pseudo-F and p-values associated with specific factor in the wild giant pandas.**

| **Factors** | **Variables (Groups)** | **Bray–Curtis** |
| --- | --- | --- |
|  |  | **pseudo-F p-value** |
| Individuals | 27(ID1/ID2/ID3/ID4/ID5/ID6/ID7/ID8/ID9/ID10/ID11/ID12/ID13/ID14/ID15/ID16/ID17/ID18/ID19/ID20/ID21/ID22/ID23/ID24/ID25/ID26/ID27) | 1.674 0.037 |
| Popultion | 2(Wolong/Baoxin) | 1.4691 0.136 |
| Collecting time | 2(2013/2015) | 1.4691 0.136 |

***number of permutations: 999**
